# Accuracy of hand localization is subject-specific and improved without performance feedback

**DOI:** 10.1038/s41598-020-76220-0

**Published:** 2020-11-05

**Authors:** Tianhe Wang, Ziyan Zhu, Inoue Kana, Yuanzheng Yu, Hao He, Kunlin Wei

**Affiliations:** 1 School of Life Sciences, Peking University, Beijing, China; 2 School of Psychological and Cognitive Sciences, Peking University, 5 Yiheyuan Road, Beijing, 100871 China; 3 Yuanpei College, Peking University, Beijing, China; 4 Beijing Key Laboratory of Behavior and Mental Health, Beijing, China; 5 Key Laboratory of Machine Perception, Ministry of Education, Beijing, China; 6 Peking-Tsinghua Center for Life Sciences, Beijing, China

**Keywords:** Human behaviour, Motor control

## Abstract

Accumulating evidence indicates that the spatial error of human's hand localization appears subject-specific. However, whether the idiosyncratic pattern persists across time with good within-subject consistency has not been adequately examined. Here we measured the hand localization map by a Visual-matching task in multiple sessions over 2 days. Interestingly, we found that participants improved their hand localization accuracy when tested repetitively without performance feedback. Importantly, despite the reduction of average error, the spatial pattern of hand localization errors remained idiosyncratic. Based on individuals' hand localization performance, a standard convolutional neural network classifier could identify participants with good accuracy. Moreover, we did not find supporting evidence that participants' baseline hand localization performance could predict their motor performance in a visual Trajectory-matching task even though both tasks require accurate mapping of hand position to visual targets in the same workspace. Using a separate experiment, we not only replicated these findings but also ruled out the possibility that performance feedback during a few familiarization trials caused the observed improvement in hand localization. We conclude that the conventional hand localization test itself, even without feedback, can improve hand localization but leave the idiosyncrasy of hand localization map unchanged.

## Introduction

Knowing the spatial position of one's hand is important for humans to maintain postures and perform actions. Both visual and proprioceptive cues are used for locating hands in space^1,2^. Though visual information plays a dominant role when both types of cues are available^3–5^, proprioception continuously updates the nervous system about the hand location. It has been found that the hand location, if informed primarily by proprioception, gradually drifts without visual calibration^6–8^. However, how the spatial pattern of hand localization errors changes over time has not been systematically investigated.

Previous studies have revealed that the localization error varies in the hand space^5,9,10^. On the group level, the localization error was affected by the distance from the body, with small localization errors in the areas close to the body and large errors in the areas away from the body^11^. The localization of the left hand was biased to the left while that of the right hand to the right^12,13^. Besides these general patterns on the group level, the spatial pattern of localization error showed large inter-individual differences^7,14^. Measured by Visual-matching tasks, the hand localization maps remained similar across conditions within a participant but differed widely across participants^3,13^. As another indirect evidence of within-subject consistency, participants also found that the localization map measured by a Visual-matching task and a pointing task was strongly correlated within a participant^15^.

However, most studies on the subject-specificity of the localization map lacked quantitative examinations of their results. Many previous studies reached their conclusions by eyeballing of data^7,14,16^. Other studies calculated the within-subject correlation coefficients between measurements from different conditions and found they were significantly larger than zero^8,17^. However, this kind of correlation results only showed the similarity between conditions as opposed to the idiosyncrasy of hand localization maps between participants. A couple of studies computed the within-subject correlation of localization maps and the between-subject correlation, but they did not compare these correlations with a statistic test, possibly due to a limited number of participants^3,13,15^. Remarkably, Kuling et al. quantitatively studied the error patterns of hand matching to visual targets or haptic targets and revealed that individual participants’ errors persisted over a month^18^. However, their matching task required the participants to reach the target in a single, uncorrected movement. This was an unconventional method for measuring capacity to estimate hand location^12,13,19^. As the participants were not allowed to adjust the positioning of their hand before registering the hand matching position, their matching errors were potentially confounded by noise and biases in action control. Thus, whether subject-specificity of localization errors persist over time needs further scrutiny.

Capacity to estimate hand position underlies motor performance in various tasks^20^. A body of studies indicated that patients with proprioception deficiency had difficulty in performing precise movements for a variety of motor tasks^21^. Loss of proprioception influences patients’ ability to control the amplitude and the onset time of movements^22^ and to coordinate the timing of different body parts^23^, resulting in substantially larger movement error^24^. With these findings, it is interesting to ask whether hand-localization capacity in healthy participants is able to predict the motor performance of the tasks that require proprioceptive control of movements. A straightforward way to test this hypothesis is to examine the relationship between the baseline accuracy of hand localization and the baseline motor performance in the same workspace.

To quantitatively study the subject-specificity of localization maps across time, here we used a Visual-matching task to measure the localization map at 100 targets in the reachable space multiple times over 2 days. We hypothesize that if localization errors are subject-specific, the localization map should be used for people identification, i.e., using one participant’s localization map measured in one measurement session to identify them from others with data in other sessions. Thus, we trained a simple classifier with the machine learning algorithm and tested its identification performance across time. We also hypothesize that the performance of hand localization measured by positioning hand to a static target could predict motor performance that requires actively locating the hand. To test this hypothesis, we measured participants’ performance in a Trajectory-matching task and correlated to their overall performance in hand localization. We found that the classifier was able to identify the participants based on the hand localization maps with an accuracy of around 70% (base rate 1/47). However, we did not find supporting evidence that baseline hand-localization performance can predict the motor performance that demands dynamically locating the hand. Surprisingly, the accuracy of hand localization improved across days, even though our measurements did not provide performance feedback. In a separate experiment, we replicated our major findings and ruled out the possibility that limited performance feedback during the familiarization trials caused the improvement in hand localization across sessions.

## Methods

### Participants

A total of forty-seven graduate students and undergraduate students (30 males, age: 21.0 ± 2.2 year, mean ± SD) of Peking University were recruited for two experiments, twenty-six for Experiment 1 and twenty-one for Experiment 2. All participants were confirmed to be right-handed by the Edinburgh handedness inventory^25^. All participants were new to the experimental task, naive to the purpose of the study, provided written informed consent before participating, and they received either course credit or monetary compensation for their time. The study was approved by the ethics committee of Peking University, and was carried out in accordance with the approved guidelines.

### Experimental setup

**Figure 1:**
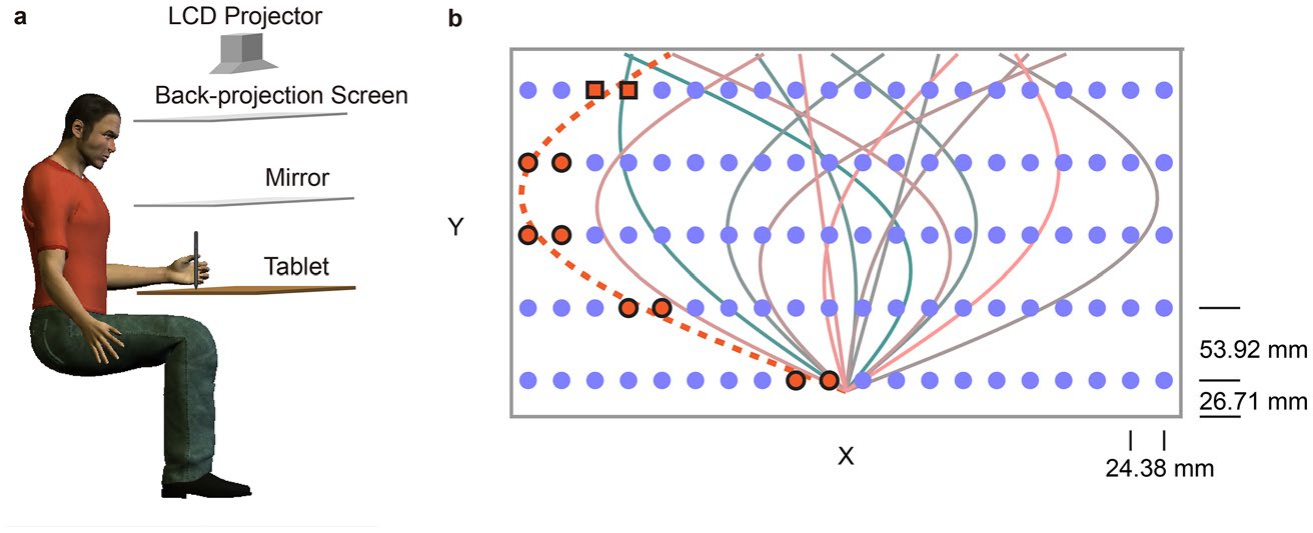
Experimental setup and material. **a** Experimental setup, **b** a schematic illustration of screen display during the experiment. Blue dots indicate the 100 targets in the Visual-matching task. Colored lines indicate the 15 target trajectories in the Trajectory-matching task. The dashed orange line and the ten orange dots illustrate how targets are chosen to quantify the local localization error for a movement trajectory. The two square orange dots denote the targets whose localization errors are chosen to compute the localization error near the endpoint of a trajectory.

The experimental setup had been used in our previous researches^26–29^. In all experiments, participants sat in front of a digitizing tablet and held the digital stylus with their left hand (Fig. 1a). They were instructed to match the tip of the stylus with either a point target or a trajectory template that was displayed on a horizontal display. The display was first projected on a back-projection screen horizontally placed above the tablet (LCD projector; Acer P1270, refreshing rate of 75 Hz). The images on the screen were then reflected by a semi-silvered mirror placed horizontally at the chest level; the reflection matched in height with the tablet where the participant’s hand was. The participants viewed the stimulus and feedback in the mirror while their view of the hand and arm was occluded. The stylus movement on the tablet was one-to-one mapped onto the visual display after calibration. Participants were required to perform the location matching as accurately as possible with their preferred pace. They also centered their body with the tablet during the whole experiment. The task was controlled by a customized program written in MATLAB (Mathworks, Natick, MA; Psychophysics Toolbox).

### Tasks

#### Visual-matching task

In each trial, a white light dot (50 mm diameter) was presented on the semi-silvered mirror to indicate the target position. The participants matched the target with the digital stylus held by the left hand. Previous studies showed that there is no fundamental difference between the proprioception of two hands^11,12^. In fact, the magnitude and spatial distribution of localization errors are similar across two hands, though the error patterns are mirror-reversed. Thus, from the perspective of measuring hand localization ability, the choice of hand does not matter. However, the motor performance is typically more variable with the non-dominant hand than with the dominant hand, especially for trajectory production^30^. One of our research goals is to examine a possible correlation between hand-localization performance (probed by Visual-matching task) and related motor performance (probed by Trajectory-matching task, see below). For better sensitivity to detect this correlation, we chose the non-dominant hand, as opposed to the dominant hand, to obtain a relatively large inter-individual variance in motor performance.

To obtain an accurate hand localization map, 100 targets were included, which formed a 5 (row) × 20 (column) matrix in the workspace in front of the seated participant (Fig. 1b). The workspace was 48.76 cm wide and 26.96 cm long, located 20 cm in front of the seated participant. The distance between the adjacent columns was 24.38 mm, and that between the adjacent rows was 53.92 mm. Each target was measured once, and the order of targets was randomized. Note, we focused on participants’ ability to localize their hand position but not movement control, so we did not ask them to make one accurate movement to hit the target. They were encouraged to adjust their hand position to match the target as accurately as possible before pressing the space bar to confirm their perceptual judgment. After the keypress, the computer speaker played a beep sound to confirm the measurement. No performance feedback was given. The target disappeared directly while the next target appeared in a new position to start the next trial. The participants were allowed to move freely from one target position to the next at their own pace. To minimize the influence of the initial hand position on reaching error^15^, we did not include a common start position in our task. Participants started their matching from the last target, so in the different measurement sessions, they started to match the same target from different positions.

Before formal data collection, 16 familiarization trials were given for the Visual-matching task. Each trial was associated with a different target, and the 16 targets were evenly spaced to form a 4 × 4 matrix to cover the whole workspace. None of them overlapped with the targets in the formal test. For these familiarization trials, the actual position of the stylus was indicated by a green dot (50 mm diameter) for one second after the participant pressed the confirmation key. The 16 targets were shown one by one from the bottom to the top and from left to right.

#### Trajectory-matching task

The Trajectory-matching task was modified from a similar task in one of our previous studies^31^. In the workspace of the Visual-matching task described above, participants were asked to produce a curved trajectory to “copy” a trajectory template that was visually presented on the projection screen (Fig. 1b). Each trial began with participants holding their left hand at a starting position indicated by a dashed circle (40 mm diameter) at the bottom center of the workspace. After 100 ms, the starting position changed from blue to green, and a beep sound was played to signal the incoming movement. Then, a trajectory template (a 20 mm-wide red line) appeared, stretching from the start position to the upper edge of the workspace. The target trajectory was prescribed by the formula: x = α × y + β × sin(πy), where y indicated the displacement in the depth direction and x indicated the displacement in the mediolateral direction. Numerically, y ranged between 0 and 1, where 1 represents 211 mm in the workspace. Thus, the main direction and curvature of the curved trajectory were determined by α and β, respectively. Participants were instructed to make a fast movement to match the target trajectory accurately. During the movement, no cursor feedback was given to show their actual hand position. After hand reaching the upper edge of the workspace, another sound was played to indicate the end of the trial. The participant returned the stylus to the starting position without cursor guidance. The hand location was only displayed as a white cursor (30 mm diameter) when it was within 5 cm around the starting position. No performance feedback was given, and a new trajectory appeared after the hand returning to the start position.

To assess participants’ performance for trajectory matching, fifteen target trajectories were included and evenly distributed over the whole workspace (Fig. 1b). These trajectories were set by varying α from − 1 to 1 and β from − 0.9 to 0.8. The absolute value of β quantified the curvature of the trajectory. All target trajectories started from the starting position at (x = 0, y = 0) and ended when y = 1. The target trajectories were presented in a random order, and each appeared twice in a row. Before the formal test, we gave each participant four trials to familiarize the task with a single target trajectory (α = 0, β = 0.1), which was not used in the formal experiment. In the first two practice trials, participants received terminal feedback by viewing the actual movement trajectory made along with the target trajectory immediately after the end of the movement. The next two practice trials were the same as the formal trial without terminal feedback.

The participant was not allowed to start a movement before the start position turned green. Moreover, no backward movement towards the body was allowed. Warning messages, i.e., “Do not move before the start position turns green” or “Do not move backward,” were shown on the screen if these trials were detected. To avoid slow movement, their average movement speed was computed on each trial and compared it to the lowest speed allowed (165 mm/s). Movements slower than this threshold were regarded as invalid, and a warning message (“Too slow”) was displayed at the trial end to urge participants to move faster. All invalid trials were repeated immediately.

### Experimental protocols

#### Experiment 1

Experiment 1 was designed to test whether the hand localization map was subject-specific and stable across days and whether it correlates to motor performance. It included three sessions with the first two sessions on day 1 and the third one on day 2. There was a 40-min rest between the first two sessions and a 24 h interval between the last two sessions. The Trajectory-matching task was performed at the end of the first Visual-matching task, and it took about 5 min. Session 1 and session 3 started with a sixteen-trial familiarization, which provided participants with feedback at the end of each trial.

#### Experiment 2

We found that accuracy in hand localization improved across sessions without any performance feedback in Experiment 1. One confounding variable was that the 16 familiarization trials before session 3 provided performance feedback, which might improve participants’ accuracy in hand localization, as shown in the subsequent measurement sessions. In Experiments 2, the familiarization trials before session 3 were removed, and an additional 4th session with its own familiarization trials was included. Other procedures remained the same as in Experiment 1. Therefore, Experiment 2 included four sessions, two on the first day and two on the second day. We were particularly interested in the Visual-matching task of session 3: the previously observed improvement in this session should be absent if it was a result of familiarization trials with feedback. Similarly, we should observe an improvement in session 4 if the familiarization trials mattered.

### Data analysis

The hand localization error direction vector (x_e_, y_e_) was defined as the differences between the location of the target position and the actual position of the stylus tip in x and y dimensions, respectively. The hand localization map was a 2 × 5 × 20 matrix, where the first dimension was the error direction vector (x_e_, y_e_) at each target position and the other two dimensions representing the coordinate dimensions (5 rows and 20 columns) of the 100 targets. The overall hand localization accuracy was quantified by the average magnitudes of the error vectors at the 100 targets in one session. As proprioception errors improved across sessions, we calculated the error reduction as the percentage difference between the first session and the other sessions by $100\mathrm{\%}\times \left({\mathrm{error}}_{1}-{\mathrm{error}}_{\mathrm{i}}\right)/{\mathrm{error}}_{1}$, where ${\mathrm{error}}_{1}$ refers to the average proprioception error of the first session and ${\mathrm{error}}_{\mathrm{i}}$ refers to that of compared sessions (i = 2, 3, 4). To compare the hand localization error in different areas, the workspace was evenly divided into left and right regions by the vertical midline, and into near and far regions by the horizontal line. Thus, the left region covered the ten columns of targets on the left, and the right region covered the other ten columns of targets on the right. The near region covered the three rows close to the participant's body, and the far region covered the other two rows away from the body.

To examine the direction of the localization error on the group-level, we averaged the error maps from different sessions for each participant and calculated the percentage of participants whose average error directing to the right and to the upward, respectively, at each target. We tested whether the percentage of participants whose error was biased to one direction was significantly different from the percentage that would be expected by chance. To do so, we performed binomial tests of the percentage against *p* = 0.5. For our sample size of *n* = 26, 18 or more participants and 8 or less participants biased to one direction (left vs. right and upward vs. downward) would lead to a significant difference.

The hand localization error map was a matrix that combined the error vectors at the 100 targets. To quantify within-subjects and between-subjects variance of hand localization maps, the Pearson correlation coefficients of the error matrices across sessions and between individuals were computed. To establish a baseline correlation between error maps, we then computed all possible pairwise correlations between every two participants for each of the three-session pairs. For example, for the correlation between session 1 and session 2, we calculated the correlation coefficients between the 1st participant’s hand localization error matrix in session 1 with the 1st–26th participants’ error matrices in session 2, and thus obtained 26 correlation coefficients. The same procedure was applied for each participant’s hand localization error matrix in session 1, resulting in 25 × 26 correlation coefficients that characterized the between-subject and between-session similarity of hand localization maps. The same kind of analysis was also applied to the Euclidean distance.

For the Trajectory-matching task, the motor error was defined by the root mean square error (RMSE) between the target trajectory and the participant’s movement trajectory. Each movement trajectory was evenly divided into 30 segments along the y-axis between the start position and the upper edge. The x-coordinate of the movement trajectory was interpolated to obtain the exact x-position at the cut points of adjacent segments. The sample rate of the measurement was 130 Hz, sufficiently high for 27.5 cm hand movements performed in about 1.2 s in our experiment. The horizontal deviation at the cut points of adjacent segments was used to compute the RMSE for each trial:

$$\mathrm{RMSE}=\sqrt{\sum_{\mathrm{y}=1}^{30}{\left({\mathrm{x}}_{\mathrm{y}}-{\mathrm{x}}_{\mathrm{y},\mathrm{t}}\right)}^{2}},$$

where ${\mathrm{x}}_{\mathrm{y}}$ and ${\mathrm{x}}_{\mathrm{y},\mathrm{t}}$ is the horizontal ordinate (x value) of the movement trajectory and the trajectory template at the cut points, respectively. For each participant, the average motor error (i.e., RMSE) and average hand localization error in session 1 and session 2 were computed. The correlations between these two baseline performance measures across participants were computed.

Since the localization errors were spatially heterogeneous but each movement trajectory only covered a small percentage of the workspace, we figured that the motor error of individual trajectories might be predicted by the localization error in its vicinity. We computed the local localization error around each trajectory by using only the localization error at the targets “sandwiched” the trajectory on each row (Fig. 1b). Thus, each trajectory would have ten local localization errors, measured in the hand-localization task. We computed the average localization error for each trajectory, and correlated it to its motor error (see below). We also wanted to test the hypothesis that subjects might only concern about the accuracy of movement endpoint, and whether the motor performance at the endpoint of the trajectory can be predicted by the hand-localization error around the endpoint. Thus, we calculated the endpoint error of each trajectory as well as the trajectory-endpoint-specific localization error, which was the average localization errors at the two closest targets “sandwiched” the trajectory endpoint. Linear regression models were built with the local error of each trajectory (or endpoint error of each trajectory) as the dependent variable, the trajectory-specific localization errors (or trajectory-endpoint-specific localization error) as the independent variable, and each participant ID (dummy variable) as the random factor. The fixed effect of the dependent variable was evaluated to examine whether these specific localization errors can predict the motor performance.

Average hand localization errors were compared between sessions or between regions by repeated-measures ANOVAs. The homoscedasticity and normality assumptions were examined before ANOVAs were performed. All dependent variables met these assumptions unless otherwise mentioned. For the data violating homoscedasticity assumptions, Greenhouse–Geisser correction was applied for ANOVAs. For the data violating normal distribution, the natural logarithm function was applied to transform the data into a normal distribution before ANOVAs. One-sample t-tests were used to compare the error reduction percentage of each session with zero. Since the average hand localization errors decreased among sessions, we then analyzed whether this decrease of error was different for different regions of the workspace (near vs. far; left vs. right). Paired t-tests were used for within-subject comparisons if normality assumptions were satisfied. Otherwise, Wilcoxon t-tests were used. Welch’s t-tests were used if equal variance assumption was violated. Correlation coefficients were submitted to Fisher’s Z transformation for between-group comparisons. Pearson correlation was default for computing linear correlations. If normality assumption was violated, Spearman’s correlation was used. Multiple t-tests and Post hoc comparisons for ANOVAs were conducted with Bonferroni corrections. All analyses were performed with MATLAB (The MathWorks, Natick, MA) and SPSS version 19 (IBM, Somers, NY). The significance level was set at *p* < 0.05.

### Convolutional neural network classifier

The convolutional neural network (CNN) algorithm was used to investigate to what extent one's hand localization map was distinguishable from the others’. We hypothesize that if the hand localization map is idiosyncratic and stable, a classifier trained by one or two sessions of hand localization map will be able to identify the individual from other individuals based on their later performance. Since the data structure of the hand localization map is a matrix similar to a digital image, our CNN classifier was constructed as a typical image classifier (Machine Learning Toolbox, MATLAB 2018b, Natick, MA). The input of the CNN classifier was a 2 × 5 × 20 hand localization error matrix. The CNN classifier contained an input layer, a convolution layer, and a normalization layer. A rectified linear unit was applied as an activation function, followed by a drop out layer and a fully connected layer. Finally, a SoftMax function was applied to change the output into the probability of each class. The kernel size of the convolution layer was 3, and the number of output filters was 13. The cross-entropy was used as the loss function, and the Stochastic Gradient Descent with momentum (SGDM) was used to optimize the CNN classifier. The initial learning rate was set at 0.01. The CNN classifier was trained for 250–500 epochs according to the size of the training set, and the input sequence was shuffled every epoch.

In Experiment 1, hand localization maps in the first two sessions from each participant served as the training set, and the maps in the third session made up the test set. In Experiment 2, session 1, 2, and 3 were used as the training set and used session 4 as the test set. Moreover, participants from both experiments were collapsed to test the classification results: all sessions in Experiment 1 and the first three sessions in Experiment 2 were used. Besides using the first two sessions (session 1 and session 2) to predict the last session (session 3), we also tried to use session 1 to predict session 3, use session 2 to predict session 3, and use session 1 to predict session 2. After training, the CNN classifier was tested by identifying a participant from all participants based on they hand localization map in the test set. The performance of the classifier was indexed by the classification accuracy, i.e., the percentage of correctly identified error maps in the test set.

### Results

#### Experiment 1

**Figure 2:**
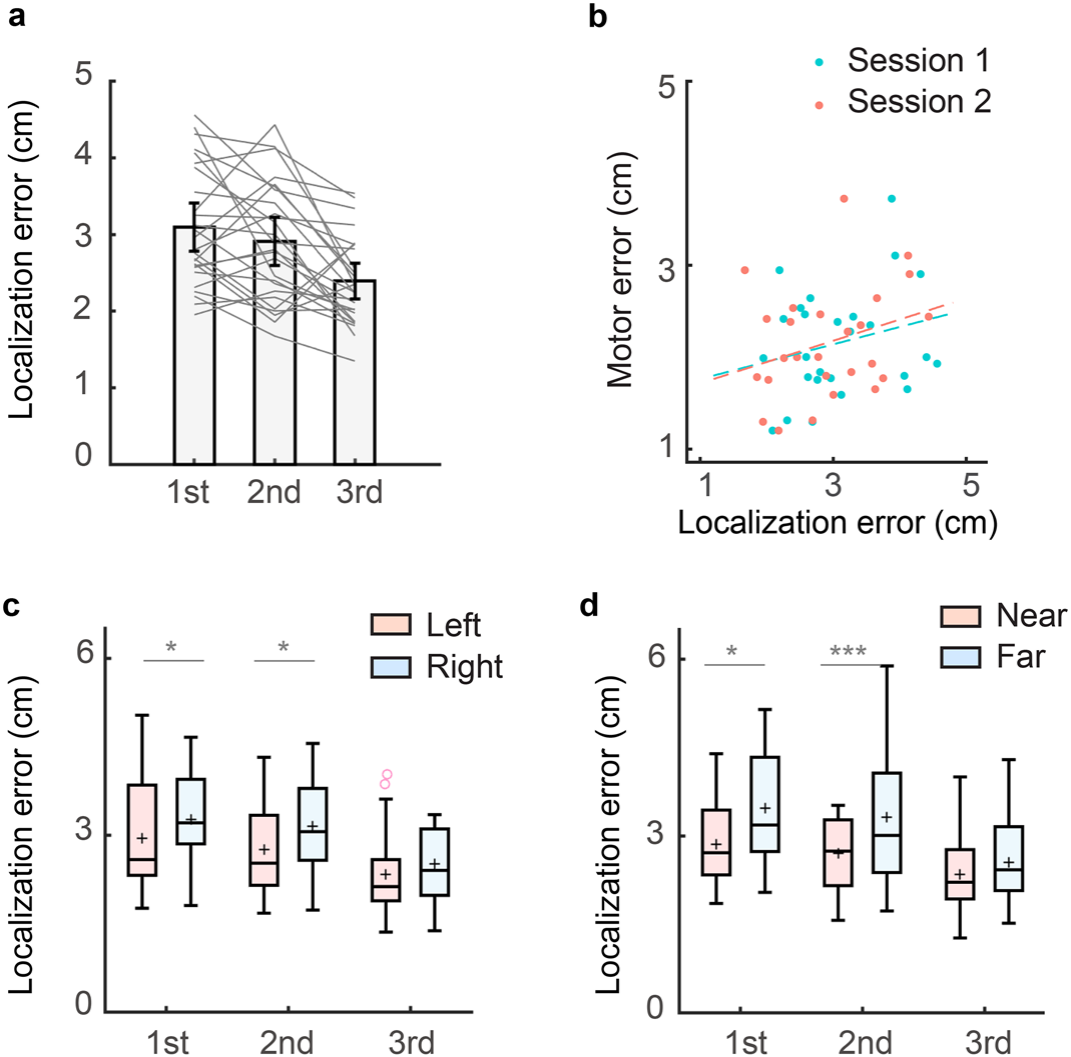
Hand localization error and motor error in Experiment 1. **a** Average hand localization error in different measurement sessions. Grey lines denote individual participants. Error bar denotes 95% confidence interval, **b** scatter plot of motor errors and hand localization errors from individual participants. The hand localization errors are plotted separately for session 1 and session 2. The dots lines indicate their corresponding linear fits, **c** average hand localization error of the left region and the right region. The band indicates the median. The box indicates the first and third quartiles and the whiskers indicate ± 1.5 × interquartile range. The black across denotes mean. The blank circle denotes outlier, **d** average hand localization error of the near region and the far regions. *p < 0.05; **p < 0.01; ***p < 0.001.

Experiment 1 aimed to examine whether the localization map is idiosyncratic and stable across a day. Interestingly, the average localization error significantly reduced over the three sessions (*F*(2,50) = 12.368, *p* < 0.001, one-way ANOVA; Fig. 2a). The average localization errors of three sessions were 3.098 cm (95% CI 2.784–3.411), 2.944 cm (95% CI 2.597–3.226), and 2.420 cm (95% CI 2.161–2.628), respectively. Post hoc pairwise comparisons indicated that the localization error of the third session was significantly smaller than that of the first session (*p* < 0.001) and the second session (*p* = 0.003). However, there was no significant difference between the first and second sessions (*p* = 0.787), which means the hand localization accuracy only improved significantly on the second day.

For the Trajectory-matching task, the average movement error was 2.172 cm (95% CI 1.915–2.400). The movement error did not correlate to the average localization error in session 1 (*r* = 0.267, *p* = 0.187; Fig. 2b) or session 2 (*r* = 0.295, *p* = 0.143). Thus, the accuracy of hand localization measured by the Visual-matching task appears not predictive of the performance of the Trajectory-matching task, though both tasks require accurate localization of the hand in the reachable space with visual targets. The motor error of different trajectories was strongly correlated with their curvatures (r = 0.886, *p* < 0.001), showing that larger curvatures led to larger RMSE. However, the motor error could not be predicted by the trajectory-specific localization errors (regression coefficient: mean = −0.015, 95% CI − 0.172 to 0.141, *t*(388) =  −0.192, *p* = 0.308). Moreover, the endpoint error of each trajectory could not be predicted by the trajectory-specific localization error (regression coefficient: mean = −0.026, 95% CI − 0.093 to 0.040), *t*(388) =  −0.773, *p* = 0.439), or the trajectory-endpoint-specific localization error (regression coefficient: mean = −0.006, 95% CI − 0.050 to 0.037, *t*(388) =  −0.284, *p* = 0.776). In sum, neither average hand-localization performance nor trajectory-specific hand-localization performance could predict the Trajectory-matching performance; hand-localization performance near the movement endpoint also failed to predict the endpoint accuracy of the Trajectory-matching task.

On the group level, the localization map showed similar spatial heterogeneity as in previous studies^5,9,10^. In the reachable workspace, the localization error was larger on the right side than on the left side. The localization error of the left region was 2.942 cm (95% CI 2.592–3.293), 2.855 cm (95% CI 2.422–3.070), and 2.363 cm (95% CI 2.058–2.603) for session 1, 2 and 3, respectively. The localization error of the right region was 3.257 cm (95% CI 2.934–3.580), 3.145 cm (95% CI 2.787–3.503), and 2.512 cm (95% CI 2.262–2.761), respectively (Fig. 2c). The localization error of the left region was significantly smaller than that of the right region in session 1 (*t*(25) =  −2.587, *p* = 0.048, paired t-test) and 2 (*t*(25) =  −2.983, *p* = 0.018, paired t-test), but not in session 3 (*t*(25) =  −1.850, *p* = 0.228, paired t-test). On the other hand, the localization error was larger on the far side of the workspace than on the near side. The localization errors of the near region were 2.859 cm (95% CI 2.573–3.146), 2.704 cm (95% CI 2.463–2.938), and 2.339 cm (95% CI 2.092–2.587) for the three sessions, respectively. The localization errors of the far region were 3.465 cm (95% CI 3.075–3.852), 3.316 cm (95% CI 2.267–3.761), and 2.549 cm (95% CI 2.267–2.827), respectively (Fig. 2d). Again, the difference between these two regions was significant in session 1 (*t*(25) =  −2.992, *p* = 0.018, paired t-test) and 2 (*t*(25) =  −5.665, *p* < 0.001, paired t-test), but not in session 3 (*t*(25) =  −1.835, *p* = 0.234, paired t-test). The improvement from session 1 to session 3 was also larger in the far region (0.916 cm, 95% CI 0.547–1.286) than in the near region (0.520 cm, 95% CI 0.210–0.830, *t*(25) = −3.506, *p* = 0.002, paired t-test). However, the improvement of the right region (0.745 cm, 95% CI 0.449–1.042) and the left region (0.612 cm, 95% CI 0.230–0.994) was not significantly different (*t*(25) =  −1.040, *p* = 0.308, paired t-test). In summary, participants performed better in the left region and in the near region when localization error was measured in the reachable workspace. The localization error decreased in all regions, while the regions with large errors had a larger decrease. This resulted in a decrease of the regional differences with improvement in localization errors over successive sessions. It is worth noting that the measurement sessions and the Trajectory-matching task did not provide any feedback about their performance. The only occasion that performance feedback was provided was the 16 familiarization trials before session 3.

**Figure 3:**
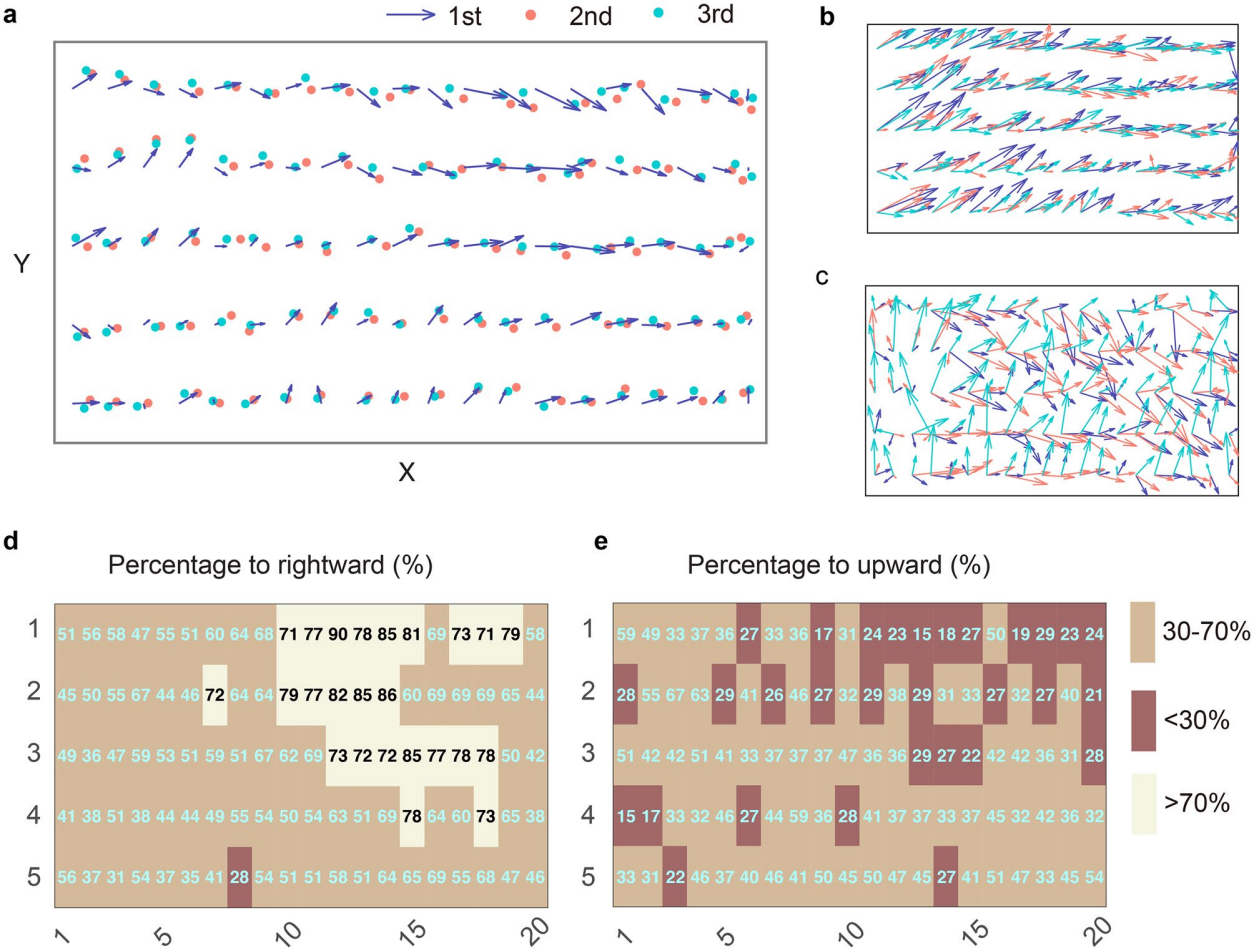
Hand localization maps the direction heatmaps on the group level. **a** Error map averaged over all participants. The purple arrow denotes the error vector of session 1 with its tail at the target location and its head at the actual hand location. The red and green dots denote the actual hand location in session 2 and 3, respectively, **b** hand localization maps from a typical participant whose error patterns remained similar across measurement sessions. The inter-session correlation coefficient was 0.68, 0.73 and 0.73 for session 1 versus 2, 2 versus 3, and 1 versus 3, respectively, **c** hand localization maps from a typical participant whose error patterns changed dramatically across sessions. The inter-session correlation coefficient was 0.58, − 0.04, − 0.08, respectively, **d** direction error in the x-dimension as a function of Visual-matching target. The number in each cell is the percentage of participants whose error directed to the right at that target. Percentages larger than 70% or smaller than 30% were significantly different from a binomial distribution with equal probabilities (n = 26), **e** direction error in the y-dimension. The number in each cell is the percentage of participants whose error directed to upward at that target.

The error vectors of all participants at 100 targets were averaged to construct a group-level localization error map (Fig. 3a). The error map of sessions 1, 2, and 3 shared a certain level of similarity. For example, the error vectors of session 1 generally pointed to the same directions as those of session 2 and 3. For all sessions, most of the error vectors pointed rightwards with larger error magnitudes when more away from the left shoulder. To examine the direction of the localization error on the group-level, we averaged the three error maps of each participant and calculated the percentage of participants whose error directing to the right at each target. Twenty-four out of one-hundred targets had rightward directional errors whose percentage of occurrence was significantly larger than the chance level of 50% (*p* < 0.05, binomial; Fig. 3d). Similarly, in the y-dimension we found 30 out of one-hundred targets had upward errors whose percentage of occurrence was significantly smaller than 50% (*p* < 0.05, binomial; Fig. 3e). Interestingly, most of these targets were clustered on the right and the far side of the workspace.

**Figure 4:**
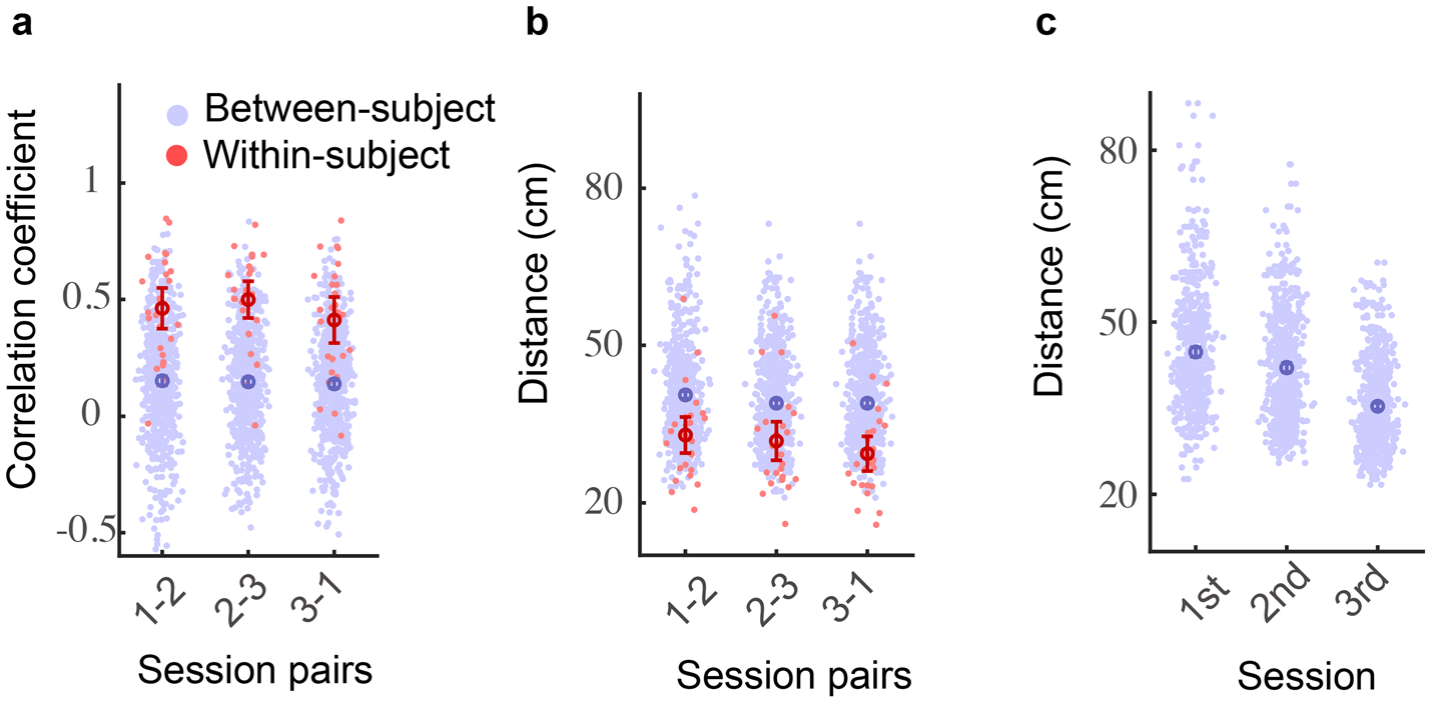
Subject-specificity of hand localization error map in Experiment 1. **a** Correlation coefficient between session pairs. Blue dotes denote between-subject coefficients. Red dotes denote within-subject coefficients. Error bars denote mean and 95% confidence interval, the same below, **b** comparisons of Euclidean distance between pairs of hand localization maps, **c** the Euclidean distance of hand localization maps from each pair of participants within a session. Each blue dot stands for a distance measured between a pair of participants, and the error bar denotes mean and 95% confidence interval.

To quantitively examine the similarity between hand localization maps, we calculated the correlation between the localization error maps of every two sessions (session 1 and 2; session 2 and 3; session 1 and 3) within each participant. The average correlation coefficients were 0.462 (95% CI 0.375–0.550), 0.499 (95% CI 0.421–0.578) and 0.412 (95% CI 0.313–0.511), respectively. Examining individual participants, we found that 25 (sessions 1 and 2), 24 (sessions 2 and 3), and 23 (sessions 1 and 3) out of the 26 participants showed significant correlations. These results indicated that the localization map remained stable across sessions for most participants (see a typical participant in Fig. 3b), and only a couple of participants showed large changes across sessions (see a typical participant in Fig. 3c). As control, the between-subject correlation coefficients were 0.153 (95% CI 0.134–0.172), 0.147 (95% CI 0.131–0.166), and 0.139 (95% CI 0.122–157) for session 1 and 2, session 2 and 3, and session 1 and 3, respectively. Importantly, for all three types of pairwise correlations, the within-subject correlation coefficients were significantly larger than the between-subject correlation coefficients (all *t*(649)s > 5, *p*s < 10^–6^, Welch’s t-tests; Fig. 4a). Thus, hand localization maps indeed demonstrated cross-session consistency within individuals.

We then analyzed the dissimilarity between hand localization maps to further evaluate the participant specificity. The within-subject and between-subject Euclidean distances between localization error maps in three session pairs were compared in the same way as the correlation coefficient. The distance between error matrices was then divided by 100 to get the average distance at a single target. The within-subject distances (mean: 0.29–0.33 cm, SD: 0.082–0.091 cm) were significantly smaller than the between-subject distances (mean: 0.39–0.41 cm, SD: 0.089–0.091 cm) of all three session pairs (all *Z*s < −4, all *p*s < 0.001, Wilcoxon t-test; Fig. 4b). In sum, localization errors remain idiosyncratic across sessions and days despite the improvement in average localization error.

We observed that the between-subject variance declined across time. The distance between the localization map of every two participants decreased across three successive sessions (*Kendall’s W* = 0.236, *p* < 0.001, Fig. 4c). Post hoc pairwise comparison showed a significant decrease between every two successive sessions (first–second: *Z* = 3.913, *p* < 0.001; second-third: *Z* = 9.391, *p* < 0.001, Wilcoxon t-test), which indicates the idiosyncratic pattern of localization map might decrease with repetitive measurements because of the improvement in average localization error.

The analysis above showed that the between-subject variance of hand localization maps was larger than the within-subject variance. However, it remains unclear to what extent one’s localization map could be distinguished from others’. Thus, we then tested whether a convolutional neural network (CNN) classifier could perform people identification based on their hand localization maps. The CNN classifier was trained for 350 echoes with the data from the first two sessions and tested with the data from session 3. The training accuracy reached 100%, and the testing accuracy reached up to 73.08% (19/26), which was substantially higher than the chance level (1/26). This means that the classifier was able to correctly identify most individuals by their performance in session 3 on day 2 when their performance on day 1 was provided. From this perspective, the spatial pattern of localization error was a person-specific feature even when it changed over time with learning.

### Experiment 2

**Figure 5:**
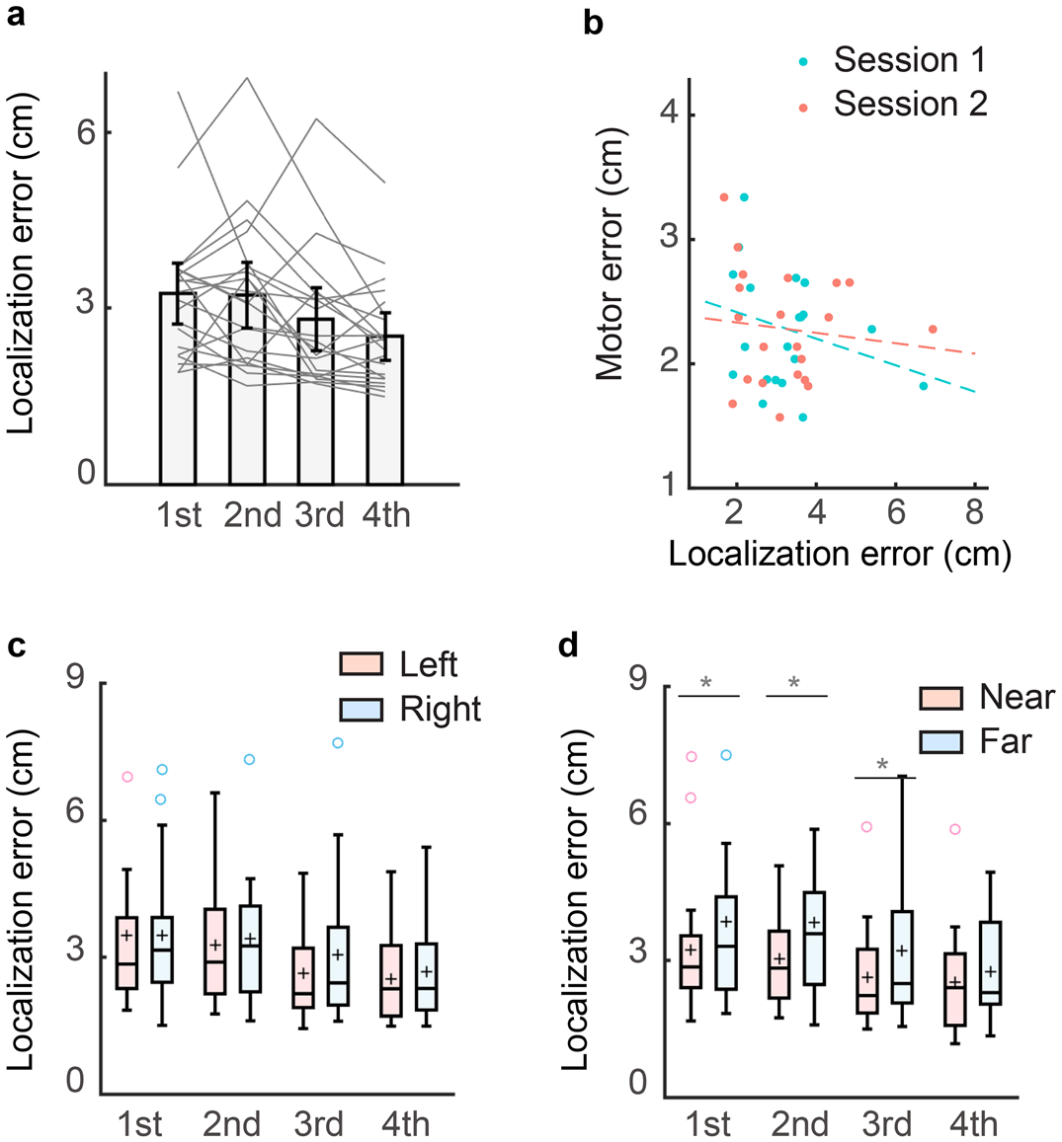
Hand localization error and motor error in Experiment 2. **a** Average hand localization error. Grey lines denote individual participants. Error bar denotes 95% confidence interval, **b** scatter plot of motor errors and hand localization errors from individual participants. The hand localization errors are plotted separately for session 1 and session 2. The dots lines indicate their corresponding linear fits, **c** average hand localization error of the left region and the right region. The band indicates the median. The box indicates the first and third quartiles and the whiskers indicate ± 1.5 × interquartile range. The black cross denotes mean. The blank circles denote outliers, **d** average hand localization error of the near region and the far regions. *p < 0.05.

In Experiment 1, we observed a significant improvement of accuracy across hand localization sessions, despite that no performance feedback was provided during the measurement. One trivial explanation is that the 16-trial familiarization with feedback before session 3 might serve as a learning session for the Visual-matching task. In Experiment 2, we thus canceled the 16-trial familiarization before session 3 to examine this possibility. On day 2, we also added another 16-trial familiarization after session 3 and before session 4 to further examine whether familiarization trials with feedback would lead to the improvement in the Visual-matching test. Consistent with Experiment 1, the hand localization accuracy improved with repetitive measurements (*Kendall’s W* = 0.288, *p* < 0.001; Fig. 5a). The error reductions in sessions 3 and 4 were significantly larger than zero (session 3: *Z* = 2.512, *p* = 0.034; session 4: *Z* = 2.485, *p* = 0.039, Wilcoxon t-test), but no significant improvement was found in session 2 (*Z* = 0.017, *p* = 1, Wilcoxon t-test). The improvement in session 3 confirmed that the improvement observed in Experiment 1 was caused by repetitive measurements as opposed to feedback-based learning in the 16 familiarization trials. Providing familiarization trials with feedback before session 4 did not further improve the performance (*Z* = 1.720, *p* = 0.512, Wilcoxon t-test), further against the possibility of feedback-based learning.

Experiment 2 also replicated other findings in Experiment 1 (Fig. 5). There was no significant correlation between the Trajectory-matching error (2.333 cm, 95% CI 2.109–2.557) and the localization error in session 1 (*r* = −0.042, *p* = −0.859, Spearman correlation, Fig. 5b) or session 2 (*r* = −0.017, *p* = 0.944, Spearman correlation). Besides, the trajectory-specific localization errors could not predict the motor error of different trajectories (regression coefficient: mean = 0.004, 95% CI − 0.024 to 0.032, *t*(298) = 0.279, *p* = 0.780). The endpoint error of each trajectory could not be predicted by the trajectory-specific localization error (regression coefficient: mean = −0.026, 95% CI −0.093 to 0.040, *t*(298) =  −0.773, *p* = 0.439), or the trajectory-endpoint-specific localization error (regression coefficient: mean = 0.014, 95% CI −0.012 to 0.039, *t*(298) = 1.048, *p* = 0.296).

Comparing average localization errors in different workspaces, we found that the means of error in the right region was larger than that in the left region in all four sessions, although none of comparisons reached significance (*p*: 0.110–0.859, Wilcoxon t-test; Fig. 5c). The error of the near region was significantly smaller than the error of the far region in the first three sessions (session 1: *Z* = −2.868, *p* = 0.045; session 2: *Z* = −2.103, *p* = 0.010; session 3: *Z* = −2.520, *p* = 0.013, Wilcoxon t-test; Fig. 5d), but not in session 4 (*Z* = −0.921, *p* = 1, Wilcoxon t-test). Similar to Experiment 1, the improvement from session 1 to session 4 was larger in the far region than in the near region (*t*(20) = 2.228, *p* = 0.038, Wilcoxon t-test), but the improvement was similar between the left region and the right region (*t*(20) =  −0.399, *p* = 0.694, Wilcoxon t-test). All these findings remained consistent with Experiment 1, although the effect size appeared smaller. This was probably because Experiment 2 has larger inter-individual differences than Experiment 1. We found larger variances of the averaged localization error in Experiment 2 when comparing its three experimental sessions to those in Experiment 1 (Session 1: *F*(20,25) = 4.0, *p* = 0.001; Session 2: *F*(20,25) = 2.6, *p* = 0.024; Session 3: *F*(20,25) = 4.2, *p* = 0.001, F-test).

**Figure 6:**
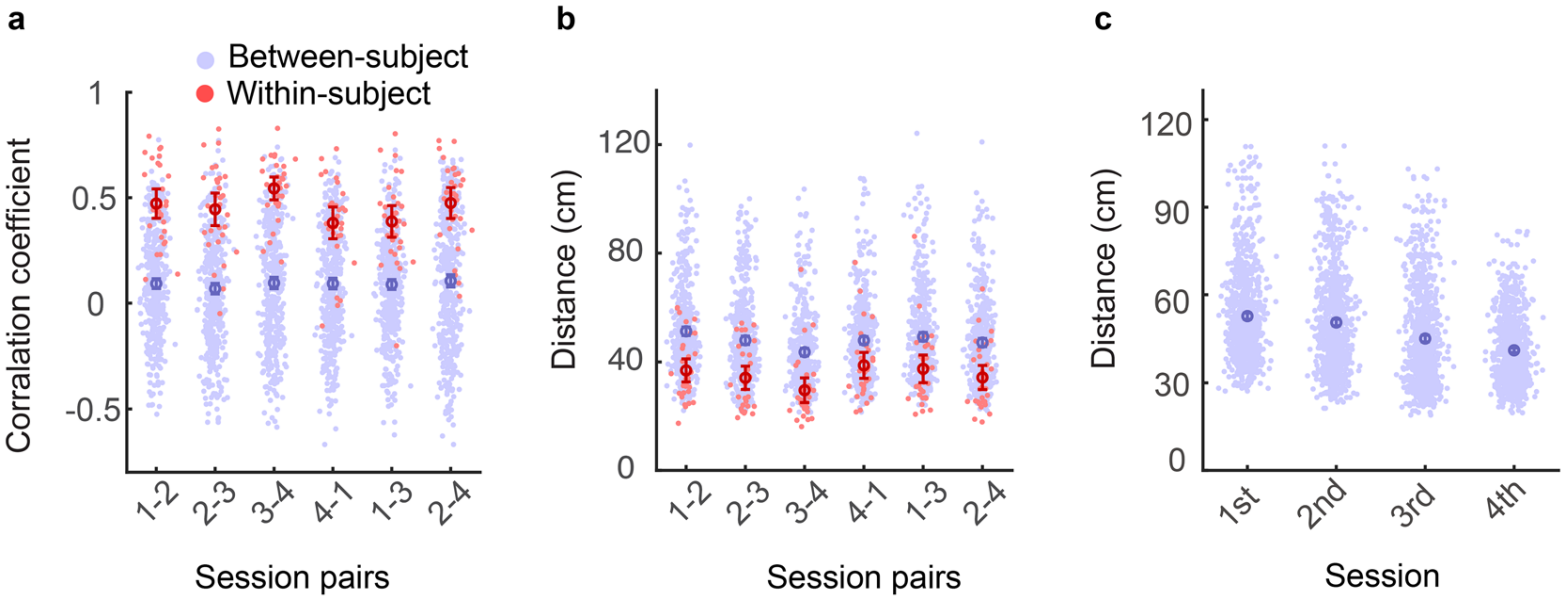
Subject-specificity of hand localization error map in Experiment 2. **a** Correlation coefficient between session pairs. Blue dotes denote between-subject coefficients. Red dotes denote within-subject coefficients. Error bars denote mean and 95% confidence interval, the same below, **b** comparisons of Euclidean distance between pairs of hand localization maps, **c** the Euclidean distance of hand localization maps from each pair of participants within a session. Each blue dot stands for a distance measured between a pair of participants, and the error bar denotes mean and 95% confidence interval.

In Experiment 2, we continued to observe that the idiosyncratic pattern of hand localization maps persisted across sessions. For the six session-pairs (session 1 vs. 2, session 2 vs. 3, session 3 vs. 4, session 1 vs. 4, session 1 vs. 3, session 2 vs. 4), the within-subject correlation coefficients between localization error matrix had a mean of 0.35–0.548 and a standard deviation of 0.161–0.260. The between-subject correlation coefficients had a mean of 0.070–0.099 and a standard deviation of 0.239–0.291. All the within-subject correlation coefficients were significantly larger than the corresponding between-subject correlation coefficients (all *t*s > 6, *p*s < 10^–5^, Welch’s t-tests, Fig. 6a). Furthermore, the within-subject distances (mean: 0.27–0.37 cm, SD: 0.079–0.126 cm) were smaller than the between-subject distances for all six comparison pairs (mean: 0.41–0.50 cm, SD: 0.13–0.17 cm, all *Z*s > 3.3, *p*s ≤ 0.005, Wilcoxon t-test, Fig. 6b). Similar to Experiment 1, the between-subject distances within each session decreased over time (*Kendall’s W* = 0.256, *p* < 0.001, Fig. 6c). Post-hoc pairwise comparisons found significant differences between session pairs of 1st–3rd, 2nd–3rd, 1st–4th, and 2nd–4th (all *p*s < 0.001). Thus, the between-subject difference between hand localization maps decreased across days but not within days.

The same CNN classifier, as in Experiment 1, was used to perform people identification based on hand localization maps. To start with, the participants' hand localization maps from session 1 and 2 made up the training set and that of session 3 as the test set. After training for 350 echoes, the classifier was able to classify the localization map from the test set with 76.19% accuracy (16/21). Then, session 1–3 were used to train the CNN classifier, and session 4 was used to test it. We obtained a 61.9% testing accuracy (13/21). We also collapsed the data from both experiments to perform people identification with 47 participants. Using hand localization maps of session 1 and 2 as the training set and third session as the test set, we obtained a testing accuracy of 72.34% (34/47). With this large dataset, we also used data from one session as the training set to predict the later sessions. The accuracy could reach 53.19% (25/47) when using session 1 to predict session 2, 55.32% (26/47) when using session 1 to predict session 3, and 61.70% (29/47) when using session 2 to predict session 3. Hence, the CNN classifier could identify individuals with a reasonable accuracy based on a single session of hand localization map. The accuracy can be further improved if an additional session of data was provided as the training data. The overall performance of people identification thus supports that hand localization maps are relatively stable and idiosyncratic among participants.

## Discussion

Whether the idiosyncratic pattern of localization map persists over time with good within-subject consistency has not been adequately investigated in previous research. We used the Visual-matching task, to repetitively measure the localization map across sessions and across days. We found that (1) the spatial pattern of localization error is subject-specific and remains idiosyncratic across days, (2) there is no supporting evidence that participants’ capacity in hand localization measured in the Visual-matching task can predict their performance in the Trajectory-matching task though both tasks demand accurate localization of the hand, (3) participants can improve their accuracy in hand localization through repetitive measurements without any performance feedback during the measurement.

It has been known for long that the error pattern of localization maps varies widely among participants^3,6,13,14^. Kuling and colleagues provided clear evidence that spatial errors during target matching tasks were systematic among individuals and persisted over time^18^. However, their measurement was a quick and uncorrected pointing task, which departed from typical measurements of hand localization accuracy^3,13,15^. After measuring hand positioning error over a large reachable space, we found that the within-subject correlation of hand localization maps between measurement sessions and days was substantially larger than the between-subject correlation. Furthermore, the within-subject dissimilarity between sessions was much smaller than the between-subject one. Remarkably, a simple CNN classifier could perform people identification based on hand localization maps with fair accuracy. These findings confirmed that the spatial pattern of the localization map indeed remains consistent over time. We postulate that subject-specific error patterns might be shaped by individuals’ unique sensorimotor experience in their lifetime since, after all, movement history^32–34^ and motor learning experience^35,36^ have considerable influence on one’s hand localization accuracy.

The improvement of hand localization accuracy without feedback was surprising at first sight. However, although feedback is considered essential for various types of learning, perceptual learning studies have reported that participants can improve their performance in visual perceptual tasks without performance feedback, such as motion-direction discrimination task^37^ and texture discrimination task^38^. Researchers even have found that the learning rate is similar to and without feedback in a direction discrimination task^39^. These perceptual improvements are generally attributed to the neural plasticity at the cellular level in the visual system^40^. We have similarly found that participants can improve their accuracy in the Visual-matching tasks with no performance feedback. This finding was observed in two different groups of participants who were tested in two separate experiments. Importantly, our Experiment 2 dropped the 16-trial familiarization trials, thus completely eliminated performance feedback between session 1 and session 3, but continued to observe the improvement of hand localization accuracy across days. We thus postulate that this observed improvement in the accuracy of hand localization a result of perceptual learning. It is possible that the accuracy improvement was caused by better motor control of hand movement, given that our participants used their non-dominant hand in the Visual-matching task and the Trajectory-matching task. We think this motor explanation unlikely for the following reasons. First, the hand-localization task was not a pointing task as in a related previous research^18^; the participant was required to carefully match their hand position with the visual target and to adjust the positioning whenever needed. Second, visual-proprioception recalibration was prevented in both tasks, given that no performance feedback was provided. Third, active hand movements without visual feedback, like the ones in our tasks, have only been shown to deteriorate the positional sense of the limb as opposed to improving it^6^.

Admittedly, familiarization of the task might contribute to the improvement in hand localization. However, we regard this potential contribution insignificant since the benefit of the familiarization typically occurs at the early phase of training. Thus, familiarization should have a large effect in session 2 on day 1. However, for both experiments, the accuracy improvement of hand localization predominately appeared on day 2 with no significant improvement in session 2 on day 1. Thus, it appears that a night of rest might be necessary for the improvement of hand localization accuracy. In fact, these findings echo similar findings in other types of perceptual learning where a rest during the night has been shown necessary. For example, in visual studies, one night of sleep is necessary for bringing a performance improvement in a texture discrimination task on the second day^41,42^. This improvement is absent if participants were deprived of REM sleep during the night^41^. Admittedly, our findings thus far cannot enable us to determine what accounts for the lack of improvement within a day, and this issue warrants further investigations.

The Visual-matching task used in the present study is a conventional method to measure proprioceptive accuracy^5,9,11,43,44^. If the measurement task itself can reduce the proprioception error, we need to consider its validity as a measurement instrument. For example, a few studies have investigated how visuomotor adaptation of reaching tasks affects proprioception of the hand^45–47^. These studies typically found a systematic bias of proprioception by comparing the measurements of the proprioception before and after visuomotor adaptation. As multiple measurements were involved, we suspect that our observed improvement across successive measurements might contaminate their data.

We found that locating the left hand was more accurate in the left workspace than in the right workspace, and in the area close to the body than away from the body. Furthermore, on the group level, participants perceived their left hand to be more left than its actual position. These spatial patterns of localization errors were consistent with previous studies^11,12^. Interestingly, the regional difference of hand localization accuracy tends to diminish over the sessions in both experiments: we observed larger improvement in the far region than in the near region to the body, closing the gap of accuracy between regions. As the overall accuracy improved, the between-subject variance of hand localization maps also decreased. Taken together, we observe a trend that improvement in hand localization accuracy reduces the heterogeneity and idiosyncrasy of hand localization maps at the same time. Whether this trend will continue with more learning sessions worths further investigations.

Our findings indicated that better accuracy in hand localization does not translate to better performance in the Trajectory-matching task. The Visual-matching task employed here to measure capacity hand localization requires participants to keep their limb stationary with respect to a reference position^6,8,43,44^. Arguably, this method can only measure participants’ ability to localize their body parts in a static state. The motor performance of our Trajectory-matching task, instead, rely on proprioception in a dynamic sense to produce an accurate movement trajectory. The ability to sense the motion of a moving effector is referred to as kinanesthesia^12^. Indeed, the accuracy of static and dynamic proprioception does not correlate well^48^ because they have different signal input channels^10,49^. Our findings thus provide behavioral evidence that individuals' performance in static proprioception might not predict their motor performance, which critically depends on accuracy in locating a moving effector. Admittedly, there is a probability that participants planned their movement by trying to match the endpoint of the trajectory. However, even when we focused on the performance at the endpoint, the hand-localization performance was still not related to the Trajectory-matching performance.

This result appears at odds with some previous findings that motor learning and proprioceptive training could benefit each other^35,36^. Wong and his colleagues showed that proprioceptive training by passively moving one’s hand around a target circle could improve the subsequent motor performance of drawing the target circle^35^. Moreover, after a brief period of motor learning, i.e., tracing a series of visual targets, participants improved their proprioceptive acuity in the same workspace of the motor learning^36^. We postulate that the mutual benefits between motor learning and proprioceptive training might be a result of their shared task features. For example, in their first study, the motor learning task required participants to grasp a handle to steer a cursor towards a visual target^35^. This task was thus similar to the Visual-matching task in which participants needed to move their hand to a visual target. Their subsequent hand localization measurement was conducted by judging the relative position of a passively located hand, which grasped the same handle, with respect to a visual target in the same workspace. Thus, both tasks involved locating the hand at the end of a movement relative to a visual target. Similarly, in their latter study, the proprioceptive training was performed by passively moving the hand by the handle to “copy” a target circle while presenting a cursor to illustrate the hand position^36^. The motor learning was performed by actively copying the same target circle. Therefore, the proprioceptive training and motor learning involved similar target trajectories and kinesthetic inputs during the movements. Thus, the mutual benefits between two types of learning might result from near transfer between similar tasks. Our observed lack of dependence between hand localization accuracy and motor performance was shown in baseline performance measurement, instead of in learning paradigms. Furthermore, our Visual-matching task was different from our Trajectory-matching task since they relied on different aspects of proprioception and involved different visual targets. We postulate that these differences thus lead to a lack of correlation in performance between the two tasks.

In conclusion, our quantitative approach demonstrates that the spatial pattern of localization error is indeed subject-specific and relatively stable across time. The idiosyncrasy of localization maps can be utilized to identify participants with fair accuracy based on individuals' performance in the Visual-matching task. Notably, we have also found that a conventional hand localization measurement, the Visual-matching task, is able to improve participants’ hand localization accuracy even when no performance feedback is given. This result suggests that extra caution should be taken in future experiments where repetitive measurements of hand localization accuracy are needed. Finally, we did not find supporting evidence that hand localization accuracy measured with static postures can predict the performance of a motor task that requires accurate positioning of a moving hand, suggesting functional independence between static proprioception and kinanesthesia.

## Data Availability

Data is available for this paper at https://osf.io/jenxa/?view_only=859b1b275cd54eafaa3df74200bdcdf1.
